# The Differential Expression of Mitochondrial Function-Associated Proteins and Antioxidant Enzymes during Bovine Herpesvirus 1 Infection: A Potential Mechanism for Virus Infection-Induced Oxidative Mitochondrial Dysfunction

**DOI:** 10.1155/2019/7072917

**Published:** 2019-03-18

**Authors:** Xiaotian Fu, Xinyi Jiang, Xinye Chen, Liqian Zhu, Gaiping Zhang

**Affiliations:** ^1^Key Laboratory for Animal Immunology of the Ministry of Agriculture, Henan Provincial Key Laboratory of Animal Immunology, Henan Academy of Agricultural Sciences, Zhengzhou, Henan Province 450002, China; ^2^College of Veterinary Medicine, Yangzhou University, Yangzhou 225009, China; ^3^Jiangsu Co-Innovation Center for Prevention and Control of Important Animal Infectious Diseases and Zoonoses, Yangzhou 225009, China; ^4^College of Animal Science and Veterinary Medicine, Henan Agricultural University, Zhengzhou, Henan Province 450000, China

## Abstract

Reactive oxidative species (ROS) are important inflammatory mediators. Electrons escaping from the mitochondrial electron transport chain (ETC) during oxidative phosphorylation (OXPHOS) in the mitochondrial respiratory chain (RC) complexes contribute to ROS production. The cellular antioxidant enzymes are important for maintaining ROS release at the physiological levels. It has been reported that BoHV-1 infection induces overproduction of ROS and oxidative mitochondrial dysfunction in cell cultures. In this study, we found that chemical interruption of RC complexes by TTFA (an inhibitor of RC complex II), NaN_3_ (an inhibitor of RC complex IV), and oligomycin A (an inhibitor of ATP synthase) consistently decreased virus productive infection, suggesting that the integral processes of RC complexes are important for the virus replication. The virus infection significantly increased the expression of subunit SDHB (succinate dehydrogenase) and MTCO1 (cytochrome c oxidase subunit I), critical components of RC complexes II and IV, respectively. The expression of antioxidant enzymes including superoxide dismutase 1 (SOD1), SOD2, catalase (CAT), and glutathione peroxidase 4 (GPX4) was differentially affected following the virus infection. The protein TFAM (transcription factor A, mitochondrial) stimulated by either nuclear respiratory factor 1 (NRF1) or NRF2 is a key regulator of mitochondrial biogenesis. Interestingly, the virus infection at the late stage (at 16 h after infection) stimulated TFAM expression but decreased the levels of both NRF1 and NRF2, indicating that virus infection activated TFAM signaling independent of either NRF1 or NRF2. Overall, this study provided evidence that BoHV-1 infection altered the expression of molecules associated with RC complexes, antioxidant enzymes, and mitochondrial biogenesis-related signaling NRF1/NRF2/TFAM, which correlated with the previous report that virus infection induces ROS overproduction and mitochondrial dysfunction.

## 1. Introduction

Bovine herpesvirus type 1 (BoHV-1) is a virus of the family *Herpesviridae* and the subfamily *Alphaherpesvirinae*. The acute virus infection causes diseases in cattle of all ages and breeds, including inflammation in the respiratory tract and reproductive system [1]. The immune suppression and mucosal damages due to the virus infection may trigger a secondary bacterial or virus infection and ultimately lead to a disease referred to as bovine respiratory disease complex (BRDC), the most important disease in cattle [2, 3]. Generally, the peak incidence of BRDC occurs in young calves of less than 6 months old, a potential reason to cause death [4]. Both BoHV-1 infection and BRDC cause great economic loss to the cattle industry worldwide [3].

Mitochondria are important cellular organelles involved in multiple functions such as energy metabolism, programmed cell death, and innate immunity. As a “powerhouse of the cell,” mitochondria play a central role in orchestrating ATP production [5]. Generally, cellular energy was provided by three metabolic pathways, that is, *β*-oxidation, tricarboxylic cycle, and oxidative phosphorylation (OXPHOS). The OXPHOS metabolic pathway, consisting of the multiprotein respiratory chain (RC) complexes I to IV and the ATP synthase (ATPase), produces about 90% of the cellular ATP [6, 7]. During OXPHOS, electrons are passing through the mitochondrial electron transport chain (ETC) in the RC complexes, and a proton gradient is established across the inner mitochondrial membrane as the energy source for ATP production. Concomitantly, electrons may escape from the ETC [8], especially at complex I, II, or III, and react with a molecular oxygen to form superoxide radical (O2^−·^), which can be converted to various components of ROS [9–11]. Therefore, electrons escaping from ETC are the main source for ROS generation. Under physiological conditions, ROS are produced at low levels and act as second messengers to regulate diverse biological processes [12], whereas excessive ROS are toxic and can damage cellular components such as lipids, proteins, nucleic acids, and carbohydrates [13, 14], while there are numerous intracellular antioxidant enzymes, such as superoxide dismutase 1 (SOD1), SOD2, glutathione peroxidase 4 (GPX4), and catalase (CAT), responsible for maintaining the redox homeostasis by disturbing ROS generation with diverse mechanisms [15].

Accumulated researches have indicated that mitochondria act as a platform for antiviral innate immune response in vertebrates, mainly depending on the activation of retinoic acid-inducible gene I- (RIG-I-) like receptor signal and the participation of the mitochondrial outer membrane adaptor protein MAVS (mitochondrial antiviral signaling protein) to protect from virus invasion [16, 17]. Therefore, in favor of viral infection or pathogenesis, mitochondria are generally damaged by the invading viruses [18]. To aid in this endeavor, viruses have developed diverse approaches to affect mitochondrial function. For example, human immunodeficiency virus-1 Tat protein reduces the mitochondria size and impairs mitochondrial fission by increasing the expression levels of fission and fusion proteins dynamin-related protein 1 (Drp1) in neurons [19], vaccinia virus inhibits OXPHOS and ETC to increase ROS production during the infection of macrophages [20], and influenza A virus can translocate viral protein PB1-F2 into the mitochondria via Tom40 channels and thereby impairs the innate immune response mediated by mitochondria [21]. For the virus HSV-1, genetically closed to BoHV-1, the viral protein US3 has been found to suppress the mitochondrial respiration by blocking ETC function [22]. BoHV-1 infection impairs mitochondria membrane potential, increases ROS levels, and reduces ATP production [23], while the mechanisms underlying these detrimental effects on the cell are not clear.

In this study, we focus on identifying the expression profiles of critical molecules in the RC complexes and antioxidant enzymes, as well as the mitochondria biogenesis-related signaling NRF1 (nuclear respiratory factor 1)/2/TFAM (mitochondrial transcription factor A signaling). Our data suggested that BoHV-1 infection increased the expression of SDHB and MTCO1, components in mitochondrial RC complexes, which is supported by the increased expression of TFAM signaling, because TFAM is a critical transcriptional regulator of mitochondrial biogenesis. In addition, we found that virus infection broadly affected the expression of the antioxidant enzymes such as SOD1, CAT, GPX4, and SOD2 at both mRNA and protein levels. The aberrant expression of certain components in the RC complexes and antioxidant enzymes as well as the NRF1/2/TFAM signaling correlated well with a previous report that virus infection stimulates excessive ROS production and mitochondrial dysfunction. These findings add our knowledge to understand the mechanisms regarding ROS production and mitochondrial damage due to the virus infection.

## 2. Materials and Methods

### 2.1. Cells and Virus

MDBK cells were maintained in DMEM (Gibco BRL) supplemented with 10% horse serum (HyClone Laboratories, Logan, UT, USA). BoHV-1 (Colorado1 stain) was propagated in MDBK cells. Aliquots of virus stocks were stored at −70°C until use. The virus was titrated in MDBK cells with results expressed as TCID_50_/mL calculated using the Reed-Muench formula.

### 2.2. Antibodies and Chemicals

The following chemical inhibitors and antibodies were used in this study: 2-thenoyltrifluoroacetone (TTFA) (Sigma, # T27006), oligomycin A (Sigma, # O4876), nicotinamide adenine dinucleotide phosphate (NADPH) oxidase (NOX) inhibitor diphenylene iodonium (DPI) (Sigma, # D2926), SOD1 polyclonal antibody (ABclonal, # A0274, 1 : 1000), SOD2 polyclonal antibody (ABclonal, # A1340, 1 : 1000), CAT polyclonal antibody (ABclonal, # A11780, 1 : 1000), GPX4 polyclonal antibody (ABclonal, # A11309, 1 : 1000), OXPHOS antibody cocktail (Abcam, # ab110413, 1 : 2000), TFAM polyclonal antibody (Thermo Fisher Scientific, # PA5-68789, 1 : 1000), NRF1 antibody (GeneTex, # PA5-40912, 1 : 1000), NRF2 antibody (Abcam, # ab137550, 1 : 500), tubulin antibody (Abcam, # ab18251, 1 : 3000), *β*-actin rabbit mAb (Cell Signaling Technology, # 4970, 1 : 2000), HRP labeled anti-mouse IgG (Cell Signaling Technology, # 7076, 1 : 3000), and HRP labeled anti-rabbit IgG (Cell Signaling Technology, # 7074, 1 : 3000).

### 2.3. Western Blot Analysis

Monolayers of MDBK cells in 60 mm dishes were infected with BoHV-1 (MOI = 1) for 2, 4, 8, and 16 hours. Cell lysates were prepared using lysis buffer (1% Triton X-100, 50 mM sodium chloride, 1 mM EDTA, 1 mM EGTA, 20 mM sodium fluoride, 20 mM sodium pyrophosphate, 1 mM phenylmethylsulfonyl fluoride, 0.5 g/mL leupeptin, 1 mM benzamidine, and 1 mM sodium orthovanadate in 20 mM Tris–HCl, pH 8.0). The respective samples were boiled in Laemmli sample buffer for 5 min, and all samples were separated on 8% or 10% SDS-PAGE. The antibodies used for Western blots were described as above. To analyze the effects that ROS had on the expression of detected proteins, MDBK cells in 60 mm dishes were pretreated with solvent DMSO or DPI (5 *μ*M) for 1 h, then infected with the virus (MOI = 1) for 16 h along with DPI (5 *μ*M) or DMSO treatment. Cell lysates prepared using the lysis buffer as described above were subjected to Western blot analysis.

### 2.4. Relative Quantification of mRNA by qRT-PCR

The total RNA was extracted from infected cells or uninfected cells using TRIzol LS Reagent (Ambion, cat: 10296010) following the manufacturer instructions. Freshly prepared total RNA (1 *μ*g) was used as a template for synthesis of first-strand cDNA with commercial random hexamer primers using the ThermoScript™ RT-PCR system kit (Invitrogen, catalogue # 11146-024) following the manufacturer instructions. The cDNA products were used as templates for real-time quantitative PCR to measure mRNA levels of indicated genes with gene-specific primers. For these studies, we analyzed SOD1 (forward primer: 5′- GTTGGAGACCTGGGCAATGT -3′ and reverse primer: 5′- TCCACCCTCGC CCAAGTCAT-3′), SOD2 (forward primer: 5′- CCCATGAAGCCTTTCTAA TCCTG-3′ and reverse primer: 5′- TTCAGAGGCGCTACTATTTCCTTC-3′), CAT (forward primer: 5′- CGCGCAGAAACCTGATGTC-3′ and reverse primer: 5′- GGAATTCTCTCCCGGTCAAAG-3′), GPX4 (forward primer: 5′- TCACCAAG TTCCTCATTGACAAGA-3′ and reverse primer: 5′- TTCTCGGAACACAG GCAACA-3′) [24], and GAPDH (forward primer: 5′- CCATGGAGAAGGCTGGGG-3′ and reverse primer: 5′- AAGTTGTCATGGATGACC-3′) [25].

Analysis of glyceraldehyde-3-phosphate dehydrogenase (GAPDH) mRNA was used as an internal control. Real-time PCR was carried out using the ABI 7500 fast real-time system (Applied Biosystems, CA). Expression levels of the tested genes were normalized to GAPDH. The relative mRNA level of each gene was calculated using the method (2^-ΔΔCT^) by comparison to the control cells.

### 2.5. Cellular ROS Assay

MDBK cells in 24-well plates were pretreated with solvent DMSO or DPI (5 *μ*M) for 1 h, then infected with BoHV-1 (MOI = 1) or mock infected with cell lysates from uninfected MDBK cells in the presence of inhibitor DPI or DMSO control for 1 h. After washing with PBS for three times, fresh medium containing DPI was added. At 16 hours after infection, the cells were washed with PBS and exposed to ROS fluorescence indicator H2DCFDA (50 *μ*M) for 30 min at 37°C. The reaction mixture was then replaced with PBS, and images were acquired under a fluorescence microscope; the fluorescence intensity of cellular ROS was quantified with software Image-pro Plus 6.

## 3. Results

### 3.1. Respiratory Complexes Are Important for BoHV-1 Productive Infection

**Figure 1:**
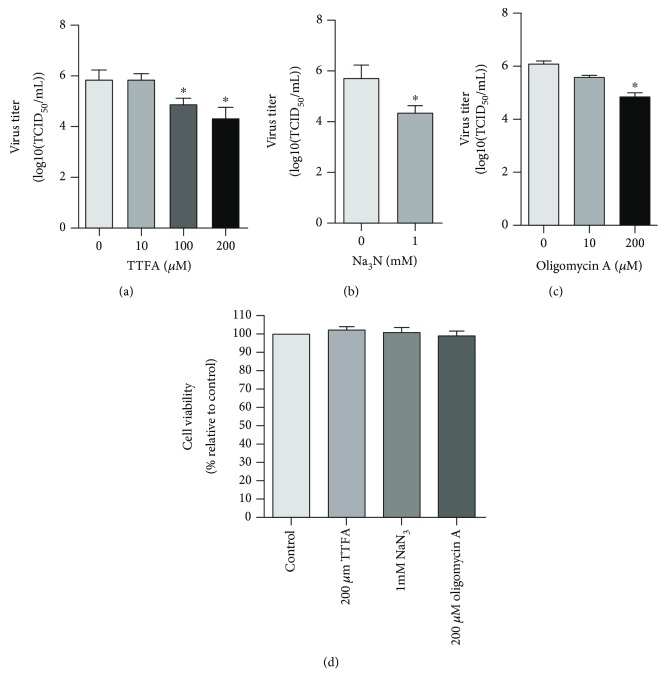
The effects of respiratory complex inhibitors on virus productive infection in MDBK cells. MDBK cells were infected with BoHV-1 (MOI = 1) for 24 h. Throughout infection, the cells were treated with TTFA (a), an inhibitor for mitochondrial RC complex II, NaN_3_ (b), an inhibitor for mitochondrial RC complex IV, and oligomycin A (c), an inhibitor for ATP synthase, at indicated concentrations, respectively. After infection, the cell cultures were subjected to frozen-thawing twice, and the viral titer was determined in MDBK cells with the results expressed as TCID_50_/mL. (d) The cytotoxicity of TTFA (200 *μ*M), NaN_3_ (1 mM), and oligomycin A (200 *μ*M) in MDBK cells for 24 h was analyzed by the Trypan-blue exclusion test as described elsewhere [47]. Data represent means of three independent experiments. Significance was assessed with the Student *t*-test. ^∗^*p* < 0.05.

We initially investigated whether chemical interruption of certain OXPHOS complexes would affect the virus productive infection by inhibitors TTFA (inhibitor for complex II), NaN_3_ (inhibitor for complex IV), and oligomycin A (inhibitor for ATP synthase, ATPase). The treatment of cells with 100 and 200 *μ*M of TTFA resulted in approximately a 1.0 and 1.7-log reduction of the virus yield compared to that in the mock-treated control, respectively (Figure 1(a)). When the virus-infected cells were treated with 1 mM of Na_3_N, the virus titer was reduced ~1.4-log compared to the mock-treated control (Figure 1(b)). Oligomycin A blocks OXPHOS by inhibition of membrane-bound mitochondrial ATPase. When the virus-infected cells were treated with oligomycin A (200 *μ*M), the virus titer was reduced ~1.2-log relative to the mock-treated control (Figure 1(c)). Of note, all the chemicals were used at a proper concentration to ensure that they did not affect cell viability (Figure 1(d)). These results suggested that integral processes in RC complexes are essential for virus productive infection.

### 3.2. BoHV-1 Infection Altered the Expression of Certain Components in Mitochondrial RC Complexes

**Figure 2:**
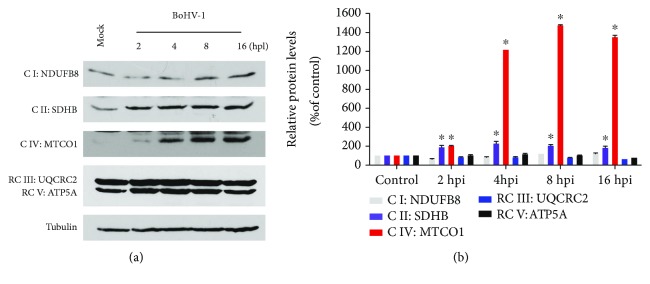
BoHV-1 infection affected the expression of certain components in mitochondrial RC complexes. (a) MDBK cells in 60 mm dishes were mock infected or infected with BoHV-1 at an MOI of 1 for 2, 4, 8, and 16 hours. The cell lysates were then prepared for Western blots to detect NDUFB8 for complex I, SDHB for complex II, MTCO1 for complex IV, UQCRC2 for complex III, and ATP5A for complex V, using OXPHOS antibody cocktail (Abcam; ab110413, 1 : 2000). Data shown are representative of three independent experiments. (b) The relative band intensity was analyzed with software ImageJ, and each analysis was compared with that of uninfected control which was arbitrarily set as 100%. Data are means of three independent experiments. Significance was assessed with the Student *t*-test (^∗^*p* < 0.05).

Next, we detect whether virus infection altered the protein expression of certain components in the mitochondrial RC complexes. Therefore, we measured the protein markers of five OXPHOS complexes by using a specific antibody cocktail against the following proteins: NDUFB8 (NADH dehydrogenase 1 beta subcomplex subunit 8) for complex I, SDHB (succinate dehydrogenase) for complex II, cytochrome c oxidase subunit I (MTCO1) for complex IV, UQCRC2 (ubiquinol-cytochrome c reductase complex 2) for complex III, and ATP5A (ATP synthase *α* subunit) for complex V. Among the detected proteins, the expressions of both SDHB and MTCO1 were significantly increased by virus infection (Figure 2(a)). Relative to the mock-infected control, the protein levels of SDHB were consistently increased ~2-fold at 2, 4, 8, and 16 hours after infection; MTCO1 was increased approximately 2-, 12-, 15-, and 14-fold at 2, 4, 8, and 16 hours after infection, respectively (Figure 2(b)), while the virus infection had no effects on the expression of NDUFB8, UQCRC2, and ATP5A (Figures 2(a) and 2(b)). These results indicated that virus infection differentially altered the expression of certain proteins in the mitochondrial RC complexes.

### 3.3. BoHV-1 Infection Differentially Affected the Expression of Certain Antioxidant Enzymes including SOD1, SOD2, CAT, and GPX4

**Figure 3:**
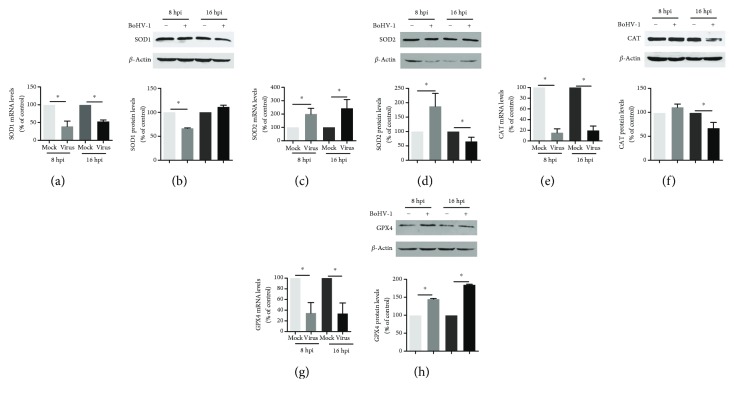
The effects of BoHV-1 infection on the gene expression of antioxidant enzymes. (a, c, e, and g) The total RNA was prepared at 8 and 16 h after infection in MDBK cells, and the mRNA levels of SOD1 (a), SOD2 (c), CAT (e), and GPX4 (g) were measured by qRT-PCR. Each analysis was compared with that of uninfected control which was arbitrarily set as 100%. Data represent three independent experiments. Significance was assessed with the Student *t*-test (^∗^*p* < 0.05). (b, d, f, and h) MDBK cells in 60 mm dishes were mock infected or infected with BoHV-1 at an MOI of 1 for 8 and 16 h. The cell lysates were then prepared for Western blots to detect the expression of SOD1 (b), SOD2 (d), CAT (f), and GPX4 (h) using SOD1 polyclonal antibody (ABclonal, #A0274, 1 : 1000), SOD2 polyclonal antibody (ABclonal, #A1340, 1 : 1000), CAT polyclonal antibody (ABclonal, #A11780, 1 : 1000), and GPX4 polyclonal antibody (ABclonal, #A11309, 1 : 1000). The band intensity was analyzed with software ImageJ. Each analysis was compared with that of uninfected control which was arbitrarily set as 100%. Data represent two independent experiments. Significance was assessed with the Student *t*-test (^∗^*p* < 0.05), ns: not significant.

Mitochondrial dysfunction is often concurrently associated with premature leaking of electrons from the ETC [26], which may ultimately lead to an increased ROS production. However, there are intracellular defense systems including the antioxidant enzymes to finely counteract ROS production [27]. Here, we initially characterized the mRNA expression of certain antioxidant enzymes including SOD1, SOD2, CAT, and GPX4 during the course of BoHV-1 infection using qRT-PCR. When MDBK cells were infected for 8 and 16 hours, the mRNA levels of SOD1, CAT, and GPX4 were unanimously decreased while SOD2 mRNA levels were significantly increased (Figures 3(a), 3(c), 3(e), and 3(g)). At 8 and 16 h after infection, relative to the uninfected control, SOD1 mRNA levels were decreased to approximately 39.2% (*p* < 0.05) and 52.3% (*p* < 0.05), respectively (Figure 3(a)); CAT mRNA levels were decreased to ~15.3% (*p* < 0.05) and 19.7% (*p* < 0.05), respectively (Figure 3(e)); GPX4 mRNA levels were decreased to ~34.3% (*p* < 0.05) and 33.4% (*p* < 0.05), respectively (Figure 3(g)); and SOD2 mRNA levels were increased to approximately 199.5% (*p* < 0.05) and 245% (*p* < 0.05), respectively (Figure 3(c)).

However, virus infection altered the steady state protein levels of these antioxidant enzymes with diverse manners which were different from that for the mRNA levels. Relative to the control, the SOD1 protein level was decreased to 66.18% (*p* < 0.05), at 8 h after infection, but at 16 h after infection, it was reversed to a level close to the control (Figure 3(b)); SOD2 protein level was increased to 186.9% (*p* < 0.05) at 8 h after infection, then decreased to 65.5% (*p* < 0.05) at 16 h after infection (Figure 3(d)); CAT protein level was decreased to 66.8% (*p* < 0.05) at 16 h after infection (Figure 3(f)); and GPX4 protein levels were consistently increased to 144.1% (*p* < 0.05) and 183.5% (*p* < 0.05) at 8 and 16 h after infection, respectively (Figure 3(h)).

**Figure 4:**
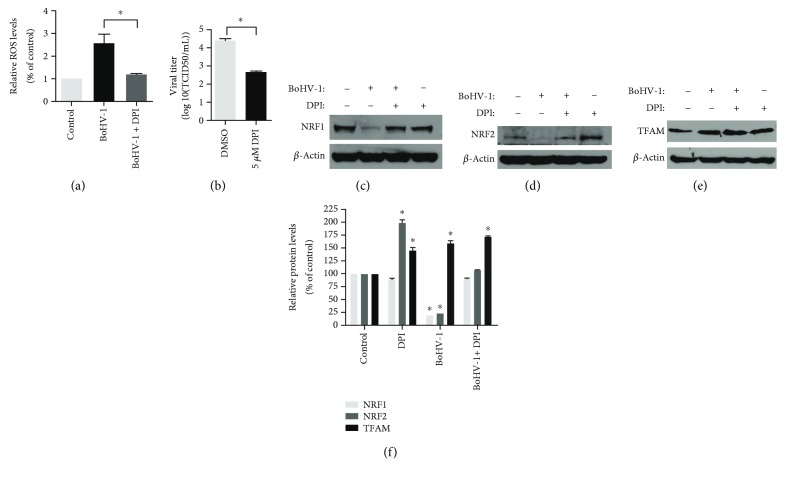
The effects of DPI treatment on the expression of NRF1, NRF2, and TFAM following BoHV-1 infection. (a) MDBK cells in 24-well plates pretreated with DPI (5 *μ*M) or DMSO control for 1 h were infected with BoHV-1 (MOI = 1) along with corresponding chemical treatment. After infection for 16 h, cellular ROS levels were determined using H2DCFDA (5 *μ*M, 30 min) (Sigma-Aldrich, St. Louis, MO, USA) and quantitatively analyzed using software Image-pro Plus 6. Data shown are means of three independent experiments. ^∗^Significant differences (*p* < 0.05), as determined by the Student *t*-test. (b) MDBK cells in 24-well plates were treated with DPI or MDSO control and infected by BoHV-1 (MOI = 1) at the same condition as described in (a). Finally, the cell cultures were subjected to frozen-thawing twice, and viral production was determined using MDBK cells with results expressed as TCID50/mL. (c–e) MDBK cells in 60 mm dishes pretreated with DPI (5 *μ*M) or DMSO control for 1 h were infected with BoHV-1 (MOI = 1) in the presence of DPI or DMSO control for 16 h; the cell lysates were prepared for Western blots to detect the expression of NRF1 (c), NRF2 (d), and TFAM (e). Data shown are representative of three independent experiments. (f) The relative band intensity was analyzed with software ImageJ, and each analysis was compared with that of the uninfected control which was arbitrarily set as 100%. Data are means of three independent experiments. Significance was assessed with the Student *t*-test (^∗^*p* < 0.05).

Taken together, the alteration of the detected antioxidant enzymes in both mRNA and protein suggested that virus infection differentially regulated the expression of these antioxidant enzymes with complicated mechanisms. The decreased protein levels of both SOD2 and CAT due to virus infection indicated a decreased capability to counteract ROS production, which is in agreement with the finding that the virus infection stimulated ROS overproduction (Figure 4(a)).

### 3.4. BoHV-1 Infection Stimulated TFAM Signaling Independent of Its Canonical Activator NRF1 and NRF2

**Figure 5:**
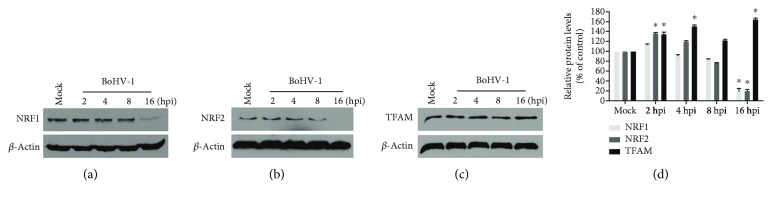
The effects of virus infection on the expression of NRF1, NRF2, and TFAM. MDBK cells in 60 mm dishes were mock infected or infected with BoHV-1 at an MOI of 1 for 2, 4, 8, and 16 hours. The cell lysates were then prepared for Western blots to detect the expression of NRF1 (a), NRF2 (b), and TFAM (c) using NRF1 antibody (GeneTex; # PA5-40912, 1 : 1000), NRF2 antibody (Abcam; # ab137550, 1 : 500), and TFAM polyclonal antibody (Thermo Fisher Scientific; # PA5-68789, 1 : 1000). (d) The relative band intensity was analyzed with software ImageJ, and each analysis was compared with that of uninfected control which was arbitrarily set as 100%. Data are means of three independent experiments. Significance was assessed with the Student *t*-test (^∗^*p* < 0.05).

TFAM signaling stimulated by transcription factor NRF1 or NRF2 is a key transcription factor regulating the expression of nuclear genes required for keeping mitochondrial respiratory function and mitochondrial DNA replication and transcription [28]. We then investigated the effects of virus infection on the expression of these transcription factors. Relative to the control, NRF1 and NRF2 were increased to approximately 114.4% (*p* > 0.05) and 136.3% (*p* < 0.05) at 2 h postinfection, then gradually decreased and peaked at 16 h after infection, which were reduced to ~19.9% (*p* < 0.05) and 19.3% (*p* < 0.05), respectively (Figures 5(a), 5(b), and 5(d)). The virus infection consistently increased the expression of TFAM from 2 h after infection and peaked at 16 h after infection, with a level increased to approximately 164.4% (*p* < 0.05) relative to the control (Figures 5(c) and 5(d)). These results indicated that the virus infection stimulated mitochondrial biogenesis-related signaling TFAM independent on its canonical upstream activators of either NRF1 or NRF2.

### 3.5. NADPH Oxidases Inhibitor DPI Affected the Expression of Both NRF1 and NRF2 But Not TFAM

NADPH oxidases (NOXs) are responsible for transporting electrons across biological membranes to generate ROS [29, 30]. BoHV-1 infection-induced excessive production of ROS is significantly decreased by NOX inhibitor DPI [9, 23, 31]. We initially detected whether ROS generation was stimulated in the context of the virus infection at an MOI of 1 for 16 hours. As expected, the intracellular ROS level was increased to ~2.6-fold (*p* < 0.05) relative to the control, which can be significantly inhibited by DPI (5 *μ*M) treatment (Figure 4(a)). In addition, DPI treatment significantly inhibited virus replication, with viral titer decreased ~1.8-log relative to the control (Figure 4(b)). In view of the fact that ROS affect various transcription factors such as nuclear factor kappa-light-chain-enhancer of activated B cells (NF-*κ*B) and activator protein-1 (AP-1) [32], we identify whether DPI treatment affected the NRF1/2/TFAM signaling at 16 h after infection. Though DPI (5 *μ*M) treatment significantly increased the expression of NRF2 but not NRF1 in the mock-infected cells, it reversed the depletion of both NRF1 and NRF2 in the virus-infected cells, which were rescued to a level almost the same as that in the uninfected control (Figures 4(c), 4(d), and 4(f)). It seems that DPI treatment affected the expression of NRF1 and NRF2 with different mechanisms. Mechanistically, ROS may negatively control the expression of NRF2 but not NRF1. The increased protein levels of TFAM in response to DPI (5 *μ*M) treatment in mock-infected cells suggest that ROS may inhibit TFAM expression (Figures 4(e) and 4(f)), while DPI (5 *μ*M) treatment did not affect the increased expression of TFAM in virus-infected cells (Figures 4(e) and 4(f)), suggesting that ROS were not involved in virus infection-induced TFAM expression.

## 4. Discussion

During coevolution with their hosts, viruses have developed sophisticated mechanisms to hijack some cellular functions for efficient infection. ATP mainly produced by mitochondria is essential not only for maintaining normal cell function but also for the viruses to complete their life cycles. Given that highly released ATP by virus-infected cells is regarded as a “danger signal” and extracellular ATP inhibits the replication of multiple viruses including HSV-1 [33], it does make sense why both BoHV-1 and HSV-1 infections decline ATP production via interruption of mitochondrial dysfunction at the late stage of infection [23, 34]. HSV-1 infection induces mitochondrial dysfunction through diverse mechanisms, e.g., in HSV-infected Vero cells, the mitochondria migrates to a perinuclear region in the cytoplasm and forms a ring-like structure, which is closely associated with viral tegument protein UL41 and UL46 [34]; HSV-1 viral protein US3 inhibits the mitochondrial RC by targeting a site between complexes II and III [22]; and HSV-1 viral protein UL7 associates with adenine nucleotide translocator 2 (ANT2) located on the inner mitochondrial membrane which is essential for exchange of cytosolic ADP for mitochondrial ATP [35, 36]. BoHV-1 infection reduced mitochondrial membrane potential (MMP) and intracellular ATP levels mediated by overproduced ROS [23]. In this study, for the first time, we provided evidence that the mitochondrial RC complexes are important for BoHV-1 infection because the widely used inhibitor TTFA for complex II, NaN_3_ for complex IV, and oligomycin A for ATPase could significantly inhibit virus productive infection (Figure 1). In addition, we found that BoHV-1 infection significantly increased the protein expression levels of subunits SDHB and MTCO1 which are important marker proteins for mitochondrial RC complexes II and IV, respectively (Figure 2). The aberrant expression of components in the mitochondrial RC complexes may partially interrupt the function of mitochondria, which supports the report that BoHV-1 infection reduced ATP production [23]. This finding provided an evidence that BoHV-1 infection disrupted mitochondrial function by affecting the expression of certain molecules in mitochondrial RC complexes.

During OXPHOS, electrons are transferred along the ETC to produce ATP. Under stress condition, a larger number of electrons will exit the ETC and generate superoxide such as O2^**−·**^, which can be converted into hydrogen peroxide (H_2_O_2_) in the mitochondrial matrix by SOD2 or in the mitochondrial intermembrane space by SOD1. H_2_O_2_ is further detoxified by antioxidant enzymes, such as by GPX4 inside the mitochondria or by the peroxisomal enzyme CAT in the cytosol [11, 26]. So, the disruption of ETC or blocking the expression of antioxidant enzymes would tend to promote ROS production. Here, we found that the virus infection significantly decreased the protein levels of both SOD2 and CAT at the late stage of infection (16 h after infection) (Figures 3(d) and 3(f)), which may compromise their capacity of converting O_2_^−·^ into H_2_O_2_ in the mitochondrial matrix as well as detoxifying H_2_O_2_ in the cytosol. These results correlated well with our finding that virus infection increases ROS production (Figure 4(a)).

It is well known that GPX4 protects the cells against oxidative stress by catalyzing the reduction of hydrogen peroxide, organic hydroperoxides, and lipid peroxides to water or corresponding alcohols at the expense of glutathione (GSH) [37]. GSH constitutes a major cellular antioxidant system and provides reducing equivalents to eliminate oxidative species [38]. BoHV-1 infection leads to a significant depletion of GSH in cell culture [39]. So, it would be disadvantageous to keep GPX4 function as normal, though virus infection apparently increased GPX4 expression (Figure 3(h)), which was consistent with the fact that virus infection induces the formation of ROS (Figure 4(a)). Ferroptosis is a mode of nonapoptotic cell death involving the production of iron-dependent ROS [40]. GPX4 has been identified as a central regulator of ferroptosis, and the inactivation of GPX4 leads to an accumulation of lipid peroxides, which consequently results in ferroptotic cell death [38, 41]. We reasoned that the increased GPX4 expression due to BoHV-1 infection would be beneficial for preventing the ferroptotic cell death to facilitate viral productive infection, which needs further study in the future.

Homeostasis of mitochondrial biogenesis is required to maintain the normal function of mitochondria, which is mainly regulated by TFAM signaling. The transcription coactivator peroxisome proliferator-activated receptor gamma coactivator 1 alpha (PGC-1*α*) is considered as the master regulator of the mitochondrial biogenesis process via interacting with two key nuclear transcription factors NRF1 and NRF2 [28, 42]. Once activated, PGC-1*α* activates NRF1 and NRF2 and subsequently downstream activator TFAM which binds to promoter regions of nuclear genes encoding certain subunits of the mitochondrial RC complexes, thereby regulating the expression of the target genes [43]. In this study, we found that BoHV-1 infection at the late stage inhibited the expression of both NRF1 and NRF2 (Figures 5(a)–5(c)). While the expression levels of TFAM, an executor of the canonical PGC-1*α* pathway, were consistently increased from 2 to 16 h after infection (Figures 5(c) and 5(d)), suggesting that virus infection promotes TFAM expression with an unknown mechanism that is independent on either NRF1 or NRF2. However, the increased expression of TFAM corroborated well with our findings that virus infection enhanced the expression of SDHB and MTCO1 (Figure 1), components in mitochondrial RC complexes.

In contrast to BoHV-1, HSV-1 infection induces TFAM depletion and consequently triggers mtDNA stress, which further stimulates the antiviral signaling and type I interferon responses [44]. So the depletion of TFAM in response to virus infection is potentially associated with priming the antiviral innate immune response. Mechanistically, the accumulation of TFAM due to BoHV-1 infection would possibly depress the antiviral innate immune response, which is an interesting issue that needs extensive study in the future.

It has been reported that the oxidants such as tertiary butyl hydroperoxide (t-BOOH), an ROS mimic, increase cellular NRF1 and TFAM gene expression in rat liver cells and hepatoma cells [45, 46]. We therefore investigated whether ROS are involved in the regulation of NRF1/2 and TFAM expressions using the NOX inhibitor DPI, which has been confirmed to block ROS production ([31] and Figure 4(a)). The enhanced expression of NRF2 but not NRF1 by DPI treatment in the mock-infected cells (Figures 4(c), 4(d), and 4(f)) indicated that ROS may partially regulate the expression of NRF2 but not NRF1. Interestingly, DPI treatment could reverse the depletion of both NRF1 and NRF2 due to virus infection (Figures 4(c), 4(d), and 4(f)), but it had no effect on the expression of TFAM (Figures 4(e) and 4(f)), which further confirmed that the virus infection stimulated TFAM signaling independent on either NRF1 or NRF2. Furthermore, DPI treatment demonstrated minor effects on the expression of TFAM in the context of virus infection, though DPI treatment led to an increased level of TFAM protein in mock-infected cells. Therefore, we assumed that certain viral components, such as viral protein and/or DNA produced during the replication cycle, rather than ROS accounted for the promoted expression of TFAM, which needs an independent investigation in the future.

In summary, our data suggest that BoHV-1 infection leads to differential expression of certain components in mitochondrial RC complexes such as SDHB and MTCO1, mitochondrial biogenesis-associated signaling of NFR1/2/TFAM pathway, and enzymes such as SOD1, SOD2, CAT, and GPX4, which correlated well with excessive ROS production and mitochondrial dysfunction. Moreover, for the first time, we found that BoHV-1 infection stimulated TFAM signaling independent of either NRF1 and NRF2 signaling or excessive ROS. These findings add our knowledge to understand the mechanisms of virus infection-induced ROS production and mitochondrial damage.

## Data Availability

The data used to support the findings of this study are available from the corresponding author upon request.
